# Compassion Satisfaction, Secondary Traumatic Stress, and Burnout among Nurses Working in Trauma Centers: A Cross-Sectional Study

**DOI:** 10.3390/ijerph18147228

**Published:** 2021-07-06

**Authors:** Hyoung Ju Lee, Miyoung Lee, Sun Joo Jang

**Affiliations:** 1 College of Nursing, Eulji University, Daejeon 34824, Korea; nurse134@naver.com (H.J.L.); mylee3730@eulji.ac.kr (M.L.); 2 Red-Cross College of Nursing, Chung-Ang University, Seoul 06974, Korea

**Keywords:** burnout, nurses, psychological, secondary traumatic stress, trauma centers

## Abstract

Due to the nature of their work, trauma nurses are exposed to traumatic situations and often experience burnout. We conducted a cross-sectional study examining compassion satisfaction, secondary traumatic stress, and burnout among trauma nurses to identify the predictors of burnout. Data were collected from 219 nurses in four trauma centers in South Korea from July to August 2019. We used the Traumatic Events Inventory to measure nurses’ traumatic experience and three Professional Quality of Life subscales to measure compassion satisfaction, secondary traumatic stress, and burnout. Multiple regression analysis confirmed that compassion satisfaction and secondary traumatic stress significantly predicted nurses’ burnout, with compassion satisfaction being the most potent predictor. The regression model explained 59.2% of the variance. Nurses with high job satisfaction, high compassion satisfaction, and low secondary traumatic stress tend to experience less burnout than their counterparts. Nurse managers should recognize that strategies to enhance job and compassion satisfaction and decrease secondary traumatic stress are required to decrease burnout among nurses in trauma centers.

## 1. Introduction

In modern society, trauma case numbers have surged due to various industrial and natural disasters, traffic accidents, and injuries, brought about as the repercussions of greater living convenience and cultural changes [1]. Trauma nurses—nurses who work in a trauma center—provide care for patients with critical trauma requiring urgent and essential treatment, such as emergency surgery and resuscitation. While providing care for these patients, trauma nurses indirectly yet strongly experience the traumatic incidents suffered by the patients [2,3,4]. Especially during the ongoing COVID-19 pandemic, society and organizations must examine the stress levels among frontline nurses, who provide care for trauma patients and promote continuous care through multiple interventions [5,6]. The study of Ruiz-Fernandez et al. [7], conducted during the COVID-19 pandemic, found that nurses working in critical situations are more likely to experience burnout.

### Background

The Diagnostic and Statistical Manual of Mental Disorders (5th Edition) defines “trauma” as an event that involves “actual or threatened death, serious injury or an equivalent threat, or extreme helplessness, fear, and terror” and includes vicarious experiences in the definition of traumatic events [8] (p. 271). Due to the nature of their work, trauma nurses feel helplessness as they witness patients’ and their families’ pain [9] and become emotionally involved as they provide care for the patients and their caregivers, who experience unexpected health crises [10]. In these circumstances, healthcare professionals tend to care for patients with compassion [11]. Compassion can be defined as the emotional sensitivity to understand other people’s suffering and combined willingness to help them to solve their problem [12]. According to the professional quality of life model [13], professionals engaged in helping others, including trauma nurses, experience compassion satisfaction (positive aspect) and compassion fatigue (negative aspect) due to working with patients who have experienced a traumatic event. Compassion fatigue consists of two components: secondary traumatic stress and burnout. Secondary traumatic stress is the stress experienced by a care provider following negative emotions resulting from witnessing a patient’s death, injury, or health-threatening event [14].

However, multiple studies have used secondary traumatic stress and compassion fatigue interchangeably and have investigated the association between compassion fatigue and burnout in emergency department nurses [15] and trauma nurses [16]. Meanwhile, some researchers have comprehensively analyzed past studies and attempted to distinguish compassion fatigue from secondary traumatic stress [17]. One such study pointed out the limitations of viewing compassion fatigue as a milder expression of secondary traumatic stress [18], and another criticized studies that equate compassion fatigue to secondary traumatic stress [19]. Compassion fatigue cannot be measured or empirically validated [18], and secondary traumatic stress and compassion fatigue should be defined and measured differently [19]. Moreover, compassion fatigue develops rapidly, whereas burnout develops slowly and lasts longer in health professionals [20]. Therefore, Stamm’s professional quality of life components should be revised from “compassion satisfaction and compassion fatigue” to “compassion satisfaction, secondary traumatic stress, and burnout”. Accordingly, in this study, based on the models of Stamm [13] and Slatten et al. [20] and following Ledoux’s [11] recommendations, secondary traumatic stress and burnout were considered as separate and independent concepts.

Trauma nurses are always exposed to stress and traumatic situations, so it can be assumed that they are at high risk of burnout [21,22]. Secondary traumatic stress is a unique phenomenon observed in professionals who deal with trauma patients. Trauma nurses, who manage high workloads [23], may experience burnout due to secondary traumatic stress, which may lead to reduced energy and various other health problems [24]. Additionally, according to recent studies, nurses who work with patients during the COVID-19 pandemic are more susceptible to secondary traumatic stress [25], which further increases their likelihood of experiencing burnout [10].

In contrast, compassion satisfaction refers to emotional satisfaction, such as the joy and pleasure derived from helping other people based on professional nursing knowledge, and the positive feeling that one can do well in their social relationships, including relationships with colleagues [13]. Compassion satisfaction can be construed as an emotional state that mitigates secondary traumatic stress; it results from helping people exposed to a traumatic event [26]. Nurses’ satisfaction and pleasure serve as a protective factor against the adverse effects of compassion fatigue [13]. Furthermore, compassion satisfaction, as a source of strength, drives nurses to continue working despite dangerous work conditions, poor patient status, and high levels of stress. As a protective factor, it can reduce and even prevent burnout [3,27].

Burnout is a multidimensional psychological syndrome that manifests due to interpersonal stressors in the work setting, such as overwhelming exhaustion, cynicism, and a lack of efficacy [28]. According to Stamm’s [13] professional quality of life model, both the environmental factors at work and individual factors can affect nurses’ burnout. Studies indicate that trauma nurses tend to experience burnout [3,4,21] and identify the following predictors of burnout: demographic characteristics (age, education, religion, marital status); work-related characteristics (workplace satisfaction, experience, work environment); personal aspects (personality characteristics); and other factors (compassion satisfaction, compassion fatigue). According to previous studies, demographic and work-related characteristics, such as age [29,30], gender [31], marital status, religion [32], education [30], and job position [30,31], can affect burnout. However, predictors identified across studies are often inconsistent and contradictory, warranting a replication study using a homogenous measurement tool [33,34]. As burnout in nurses increases turnover intention [28] and can compromise the quality of nursing care and patient safety [35], efforts to prevent or alleviate it by identifying factors that influence burnout are needed at the management level [36]. Thus, presumably, the environmental factors at work, nurses’ individual factors, nurses’ compassion satisfaction, and secondary traumatic stress can be predictors of trauma nurses’ burnout. Therefore, in this study we aimed to examine trauma nurses’ traumatic event experience, determine their compassion satisfaction, secondary traumatic stress, and burnout, and identify the predictors of burnout to provide foundational data to inform the development of prevention and intervention strategies targeting burnout among trauma nurses.

## 2. Materials and Methods

### 2.1. Design and Participants

This study was a cross-sectional investigation of traumatic event experience, compassion satisfaction, secondary traumatic stress, and burnout in trauma nurses. A total of 219 nurses directly involved in patient care in the trauma bay, traumatic ICU, or trauma ward in four nationally approved trauma centers in South Korea were included. The inclusion criteria were as follows: (1) nurses working in trauma centers, (2) nurses providing care for trauma patients for over six months, and (3) nurses who understood this study’s purpose and signed the written informed consent voluntarily. The exclusion criteria were: (1) nurses who were working in trauma centers but were not directly working with the trauma patients (e.g., physician assistants, unit managers, and administrative nurses) and (2) nurses who had less than six months of experience with trauma patients.

### 2.2. Data Collection and Ethical Considerations

The sample size was determined using G-power 3.1.9.4 software for regression analysis [37]. For a multiple regression using a two-tailed test with an effect size (f) of 0.15, a significance level (α) of 0.05, power (1-β) of 0.95, and 18 predictive variables, the minimum required sample size was 214. Assuming a 10% withdrawal rate, the questionnaire was distributed to 238 potential participants, of whom 225 returned it. We used the convenience sampling method and contacted the nursing divisions of each hospital that was the region’s designated trauma center for permission to collect data. The participants received an information sheet explaining the purpose of the study and guaranteeing anonymity along with a written consent form. They were informed that they could withdraw from the study at any time and that the collected data would only be used for research purposes. Those who signed the consent form were instructed to complete the self-reported questionnaire on their own. Completed questionnaires were placed in an anonymous envelope and collected in person or via mail. After excluding six incomplete questionnaires, 219 questionnaires were included in the final analysis. This study was conducted according to the guidelines of the Declaration of Helsinki and approved by our local institutional review board.

### 2.3. Measures

#### 2.3.1. Traumatic Event Experience

Traumatic event experience was assessed using the Traumatic Event Inventory, a 13-item tool [38] that measures the number of times that nurses experienced the same traumatic event that a patient they are taking care of has experienced during the past month, through a five-point Likert scale ranging from “rarely” (1) to “very frequently” (5). The total score ranges from 13 to 65. A higher score indicates a more severe traumatic event experience. Cronbach’s α was 0.90 at the time of development [38] and 0.81 in this study. Six trauma professionals verified this scale’s content validity. The scale’s content validity index/average for each item was 0.98, and the scale’s content validity index/universal for each item was 0.86.

#### 2.3.2. Professional Quality of Life

The professional quality of life model consists of two main constructs: compassion satisfaction (positive aspect) and compassion fatigue (negative aspect), with compassion fatigue further consisting of two components: secondary traumatic stress and burnout [13]. Compassion satisfaction, secondary traumatic stress, and burnout were measured using the Professional Quality of Life (ProQOL) Version 5 [13], which has three independent subscales and has been culturally adapted for use in several countries, including Korea. Each language version of the ProQOL is publicly available on the official website [39]. Many studies on nurses have utilized the subscales of the ProQOL Version 5 to measure compassion satisfaction, secondary traumatic stress, and burnout independently [3,16,32,40]. The tool comprises 30 items, with 10 items each on compassion satisfaction, secondary traumatic stress, and burnout, and each item is rated on a five-point Likert scale ranging from “strongly disagree” (1) to “strongly agree” (5). The total score range is 10–50; scores are classified as low (≤22), moderate (23–41), and high (≥42), and higher scores indicate higher compassion satisfaction, secondary traumatic stress, and burnout. As suggested by the developer, the total score was standardized to a t-score with a mean of 50 ± 10. The standardized scores were classified as low (<25%), moderate (25–74%), and high (≥75%), with higher scores indicating a higher frequency of compassion satisfaction, secondary traumatic stress, and burnout. The construct validity was assessed using confirmatory factor analysis [41] and goodness-of-fit indices (comparative fit index = 0.95, Tucker Lewis index = 0.95, root mean square error of approximation = 0.06). ProQOL Version 5′s Cronbach’s α for compassion satisfaction and secondary traumatic stress were 0.88 and 0.81 at the time of development and 0.90 and 0.78 in this study, respectively. Additionally, ProQOL Version 5′s Cronbach’s α for burnout was 0.75 at the time of development and 0.68 in this study.

### 2.4. Data Analysis

Data were analyzed using SPSS/WIN25.0 software. Participants’ general characteristics, work-related characteristics, traumatic event experience, compassion satisfaction, secondary traumatic stress, and burnout were analyzed using descriptive statistics. Differences in burnout according to general characteristics and work-related characteristics were analyzed using *t*-tests, χ^2^ tests, and ANOVA, followed by Scheffé’s test for post hoc comparisons. Correlations between age, clinical career length, traumatic event experience, compassion satisfaction, secondary traumatic stress, and burnout were analyzed using Pearson’s correlation coefficients. Predictors of burnout were analyzed using multiple regression, and to examine the goodness of fit of the regression model, normality and equal variance were tested using the Kolmogorov-Smirnov test and the Breusch-Pagan test, respectively.

## 3. Results

### 3.1. General and Work-Related Characteristics

Table 1 shows the participants’ general and work-related characteristics. The mean age was 27.52 ± 4.75 years (age range = 22–46 years, median age = 26 years); 87.7% of the 219 participants were female. A total of 87.2% were single, and 64.4% had no religion. The most common educational level was a bachelor’s degree in nursing (79.0%), and the nursing career length was 1–2 years (42.5%). Regarding the job position, 89.0% were staff nurses, whereas 11.0% (24 participants) were charge nurses or higher. Job satisfaction was “moderate” in 50.7% of the nurses, and 75.3% of the nurses specified that they wanted to continue working in a trauma center.

### 3.2. Traumatic Event Experience, Compassion Satisfaction, Secondary Traumatic Stress, and Burnout

Table 2 summarizes participants’ traumatic event experiences. Trauma nurses most commonly experienced a physical injury from a fall. The mean total traumatic event experience score was 34.19 ± 7.14, and the mean rating was 2.62 ± 0.55. The mean scores for compassion satisfaction, secondary traumatic stress, and burnout were 32.36 ± 5.76, 25.79 ± 5.34, and 27.28 ± 4.47, respectively; scores were classified as high, moderate, and low, as shown in Table 3. Of the participants, 4.6% had high compassion satisfaction, 0% had high secondary traumatic stress, and 0.5% had high burnout scores. Furthermore, when the scores were standardized to identify high-risk groups, 21.0% of nurses were in the low compassion satisfaction group, whereas 50.7% and 59.8% were in the moderate or high groups for secondary traumatic stress and burnout, respectively.

### 3.3. Burnout According to General and Work-Related Characteristics

Table 4 shows the differences in burnout according to participants’ general and work-related characteristics. Burnout significantly differed according to age, education level, job position, job satisfaction at trauma center, and intent to continue working at a trauma center.

### 3.4. Correlations Between Traumatic Event Experience, Compassion Satisfaction, Secondary Traumatic Stress, and Burnout

Age was positively correlated with total clinical career length and compassion satisfaction and negatively correlated with burnout. Number of working years was positively correlated with compassion satisfaction and negatively correlated with burnout. There was a positive correlation between traumatic event experience and compassion satisfaction, a negative correlation between compassion satisfaction and burnout, and a positive correlation between secondary traumatic stress and burnout (Table 5).

### 3.5. Predictors of Burnout

To identify predictors of burnout, multiple regression was performed via the enter method (Table 6). The Durbin-Watson index was 1.91, confirming that the dependent variable was not autocorrelated. The variance influence factor was below 10, confirming the absence of multicollinearity among the independent variables. The residual analysis also confirmed the fit of the regression model and the assumption of equal variance. Multiple regression analysis revealed that compassion satisfaction was the most potent predictor of burnout, followed by secondary traumatic stress and job satisfaction at trauma center; combined, these variables explained 59.2% of the variance in burnout. The effect size in this study (f^2^ = 0.28) was calculated based on the formula of Cohen [42] and approached the value denoting a large effect size (f^2^ = 0.35) [43].

## 4. Discussion

In this analysis of the differences in burnout according to nurses’ individual and work-related characteristics, the degree of burnout was significantly higher among those under 30 years [29,30]. Furthermore, nurses with a master’s degree and staff nurses had higher burnout scores; this finding is consistent with that from Jang and Kim’s study [30]. Additionally, the present results showed that burnout did not significantly differ according to gender, marital status, and religion, which is consistent with previous results [7,30,32,44]. In particular, the regression analysis of Polat et al. [32] confirmed that nurses’ spiritual orientation does not influence burnout. However, our results contradict the finding that married nurses in tertiary hospitals exhibit lower burnout [45] and the results for trauma nurses ascertained by Higgins et al. [3] and Cook et al. [21]. This may be attributable to the lower mean age and lower proportion of married nurses among our participants and to the special nature of a trauma center. Furthermore, the degree of burnout was significantly higher among those who were dissatisfied with their job and those who did not intend to continue working at a trauma center. This is consistent with the results of Lee and Kim [36] and Wang et al. [45], indicating that nurses who are dissatisfied with their current workplace and who do not intend to continue working there have a high degree of burnout and, thus, report a high turnover intention.

There were no significant differences in burnout based on the length of clinical career and type of nurse unit, consistent with the study of Ruiz-Fernandez et al. [7], which was conducted during the COVID-19 pandemic. Furthermore, there was a mild negative correlation between the length of employment and burnout. In previous studies on pediatric hospital nurses [46] and oncology nurses [47], burnout increased with the increasing length of career in direct care. Furthermore, in a study on nurses working in the public health system during the COVID-19 pandemic, there was no significant relationship between working years and burnout [7]. This discrepancy seems to stem from the differences in the target patients of care and in the limiting criteria for total clinical career and direct care. Future studies should examine the level of burnout according to working years.

On a 100-point scale, the traumatic event experience score was 52.6 and the secondary traumatic stress score was 52.0, showing that traumatic events are a prevalent phenomenon among trauma nurses [34,48]. In this study, the experience of traumatic events was measured using a tool that assessed the frequency and severity of the traumatic event [38]; however, traumatic event experience was not found to be significantly correlated with burnout, and it did not impact the level of burnout among nurses. It can be understood then that how the traumatic event is interpreted and accepted by the individual has an influence on burnout, rather than the traumatic event itself. The result that burnout decreased with increasing compassion satisfaction was consistent with previous findings on nurses in other clinical settings [26,40,49], which confirms the finding that compassion satisfaction buffers or moderates compassion fatigue, the latter of which has an adverse impact on nurses [12]. There was no significant correlation between compassion satisfaction and secondary traumatic stress. Although this finding was consistent with the results of Durkin et al. [27], it contradicted the results of Zakeri et al. [40], which revealed a strong negative correlation between the two variables in a clinical nurse population. The positive correlation found between secondary traumatic stress and burnout was consistent with previous findings [4,26,40,49].

We conducted a regression analysis to identify the predictors of burnout in trauma nurses. Satisfaction with the current unit, compassion satisfaction, and secondary traumatic stress were the only factors that significantly affected burnout in trauma nurses. This is in line with previous results showing that compassion satisfaction and secondary traumatic stress are potent predictors of burnout [40], that secondary traumatic stress predicts burnout [50], that job satisfaction is associated with nurses’ burnout [51,52], and that the work-life characteristics of a trauma center affect burnout in trauma nurses [53]. Additionally, age, education level, job position, intent to continue working, and traumatic event experience did not significantly predict burnout. This can be viewed in the same context as the findings of Lee and Kim [36], which indicated that general characteristics such as age, religion, and marital status as well as job-related characteristics did not influence turnover intention when other variables were controlled.

According to Trumello et al. [6], nurses working with COVID-19 patients experienced lower compassion satisfaction, higher secondary traumatic stress, and higher burnout compared to their counterparts who do not work with COVID-19 patients. Recently, intervention studies have been conducted to reduce burnout in nurses by improving compassion satisfaction and lowering secondary traumatic stress [54,55]; all the interventions had positive effects in reducing burnout. Furthermore, Yu et al. [47] reported that psychological adjustment training and training related to patient death reduced burnout, calling for further studies to explore measures and develop interventions to prevent and manage burnout in trauma nurses. Thus, future interventions that aim to reduce the secondary traumatic stress trauma nurses experience are necessary. Further, enhancing compassion satisfaction may help prevent burnout in trauma nurses.

This study has some limitations. First, the mean age was 27.52, and 82.7% of the participants were under 30 years. According to the World Health Organization [56], approximately 50% of all nurses in countries in the Western Pacific region are relatively young, with less than 10 years of experience; thus, we must address this point as a limitation in this study. Second, because only four out of the 17 trauma centers in South Korea were selected, selection bias was inevitable, limiting the findings’ generalizability. Third, ProQOL’s burnout subscale’s reliability is relatively low in this study. As Hemsworth et al. [26] suggested, the ProQOL’s three subscales—compassion satisfaction, secondary traumatic stress, and burnout—should be revised to increase its reliability and validity. Furthermore, this tool was adapted for use in Korea 10 years ago; therefore, it may not fully reflect the rapidly changing modern society and the trauma nursing environment (i.e., the COVID-19 pandemic). Hence, ProQOL Version 5 should be reexamined to ensure better and more rigorous cultural adaptation. Additionally, the nature of a cross-sectional study limits conclusions on causality. Therefore, we suggest subsequent studies to address these limitations by using random sampling and a longitudinal design to identify the predictors of burnout in trauma nurses.

## 5. Conclusions

This cross-sectional study attempted to examine the degree of traumatic event experience and burnout and to identify predictors of burnout among trauma nurses, with the ultimate goal of laying a foundation for developing interventions to prevent burnout in trauma nurses. The results showed that job position, satisfaction with current nurse unit, compassion satisfaction, and secondary traumatic stress influence trauma nurses’ burnout, with compassion satisfaction being the most potent predictor. Nursing managers should recognize that strategies to enhance job and compassion satisfaction and to decrease secondary traumatic stress are required to decrease burnout among nurses in trauma centers. Subsequent studies should explore strategies to improve compassion satisfaction and measures to lower secondary traumatic stress and boost job satisfaction at trauma centers to manage and prevent burnout in trauma nurses.

## Figures and Tables

**Table 1:** Participants’ general characteristics and work-related characteristics (N = 219).

Characteristic	Category	*N*	*%*	*M*	(*SD*)
Gender	Female	192	87.7		
	Male	27	12.3		
Age (years)	≤25	88	40.2	27.52	4.75
	26–30	93	42.5		
	≥31	38	17.4		
Marital status	Single	191	87.2		
	Married	28	12.8		
Religion	Christian	40	18.3		
	Buddhist	17	7.8		
	Catholic	21	9.6		
	None	141	64.4		
Education level	Three-year college	24	11.0		
	Four-year college	173	79.0		
	≥Graduate school	22	10.0		
Total clinical career (years)	<1	11	5.0	2.72	0.89
	1–2	93	42.5		
	3–5	62	28.3		
	≥6	53	24.2		
Job position	Staff nurse	195	89.0		
	≥Charge nurse	24	11.0		
Unit	TER	48	21.9		
	TICU	146	66.7		
	Trauma ward	25	11.4		
Job satisfaction at trauma center	Dissatisfied	23	10.5		
	Moderately satisfied	111	50.7		
	Satisfied	85	38.8		
Wish to continue working in a trauma center	Yes	165	75.3		
	No	54	24.7		

Note: M = mean; SD = standard deviation; TER = Trauma Emergency Room; TICU = Trauma Intensive Care Unit.

**Table 2:** Frequency of traumatic event experience among participants (N = 219).

	Traumatic Event	*M*	*SD*	Min-Max
1	Serious physical injury from a traffic accident	4.30	0.77	1–5
2	Physical injury from a fire or gas explosion	1.88	0.88	1–5
3	Physical injury from a collapse of building or installation	2.61	1.23	1–5
4	Physical injury from a machine	3.49	1.03	1–5
5	Physical injury due to a fall from a high place	4.43	0.77	1–5
6	Physical injury related to a natural disaster	1.33	0.61	1–5
7	Physical violence and abuse by family	2.11	0.98	1–5
8	Sexual violence or abuse by family	1.46	0.76	1–5
9	Physical violence and abuse by others	3.08	1.05	1–5
10	Sexual violence or abuse by others	1.54	0.80	1–5
11	Death by murder, accidents, or incidents	2.02	1.20	1–5
12	Serious physical injury from a suicide attempt	3.49	1.06	1–5
13	Death by suicide	1.03	0.18	1–5
Total		34.19	7.14	13–54
Mean rating		2.62	0.55	1–5

Note: M = mean; SD = standard deviation; Min = minimum; Max = maximum.

**Table 3:** Degree of compassion satisfaction, secondary traumatic stress, and burnout (N = 219).

Variables	Score Category	*N*	*%*	*t*-Score Category	*N*	%	Mean Rating		Total Score		Min-Max
							*M*	*SD*	*M*	*SD*	
CS	High (≥42)	10	4..6	High (≥75%)	60	27.4	3.24	0.58	32.36	5.76	17–50
	Moderate (23–41)	194	88.6	Moderate (25–74%)	113	51.6					
	Low (<23)	15	6.8	Low (<25%)	46	21.0					
SS	High (≥42)	0	0.0	High (≥75%)	60	27.4	2.56	0.53	25.79	5.34	14–41
	Moderate (23–41)	154	70.3	Moderate (25–74%)	51	23.3					
	Low (<23)	65	29.7	Low (<25%)	108	49.3					
BO	High (≥42)	1	0.5	High (≥75%)	56	25.6	2.73	0.45	27.28	4.47	13–43
	Moderate (23–41)	178	81.3	Moderate (25–74%)	75	34.2					
	Low (<23)	40	18.3	Low (<25%)	88	40.2					

Note: CS = compassion satisfaction; SS = secondary traumatic stress; BO = burnout; M = mean; SD = standard deviation.

**Table 4:** Degree of burnout according to general characteristics and work-related characteristics (N = 219).

Characteristic	Category	*N*	*M*	*SD*	*t*/*F*	*p*
Gender	Female	192	27.44	4.45	1.45	0.148
	Male	27	26.11	4.51		
Age	≤25 ^a^	88	28.02	4.44	4.98	0.008 **
	26–30 ^b^	93	27.37	4.30		a,b > c
	≥31 ^c^	38	25.34	4.50		
Marital status	Single	191	27.47	4.38	1.67	0.096
	Married	28	25.96	4.96		
Religion	Christian	40	26.65	4.85	0.43	0.731
	Buddhist	17	27.94	5.76		
	Catholic	21	27.62	4.21		
	None	141	27.33	4.25		
Education level	Three-year college ^a^	24	29.13	4.26	6.83	0.001 **
	Four-year college ^b^	173	27.38	4.24		a,b > c
	≥Graduate school ^c^	22	24.45	5.28		
Job position	Staff nurse	195	27.55	4.28	2.58	0.010 *
	≥Charge nurse	24	25.28	5.44		
Total clinical career (years)	<1	11	26.18	2.64	1.64	0.182
	1–2	93	27.78	4.93		
	3–5	62	27.58	4.10		
	≥6	53	26.26	4.23		
Unit	TER	48	26.69	4.43	1.82	0.165
	TICU	146	27.22	4.36		
	Trauma ward	25	28.76	5.02		
Job satisfaction at trauma center	Dissatisfied ^a^	23	30.22	4.53	10.33	<0.001 ***
	Moderately satisfied ^b^	111	27.72	4.17		a > b,c
	Satisfied ^c^	85	25.91	4.38		
Wish to continue working in a trauma center	Yes	165	26.85	4.28	2.52	0.013 *
	No	54	28.59	4.80		

Note: * *p* < 0.05, ** *p* < 0.01, *** *p* < 0.001. M = mean; SD = standard deviation; TER = Trauma Emergency Room; TICU = Trauma Intensive Care Unit.

**Table 5:** Correlations between age, career length, traumatic event experience, compassion satisfaction, secondary traumatic stress, and burnout (N = 219).

Characteristics	Age	Total Clinical Career	TE	CS	SS	BO
	*r* (*p*)	*r* (*p*)	*r*(*p*)	*r* (*p*)	*r* (*p*)	*r* (*p*)
Age	1					
Total clinical career	0.96 (<0.001) ***	1				
TE	0.13 (0.052)	0.12 (0.082)	1			
CS	0.31 (<0.001) ***	0.29 (<0.001) ***	0.16 (0.022) *	1		
SS	−0.03 (0.718)	0.01 (0.890)	0.07 (0.323)	0.11 (0.121)	1	
BO	−0.23 (0.001) **	−0.19 (0.004) **	−0.01 (0.862)	−0.60 (<0.001) ***	0.37 (<0.001) ***	1

Note: * *p* < 0.05, ** *p* < 0.01, *** *p* < 0.001. TE = traumatic event experience; CS = compassion satisfaction; SS = secondary traumatic stress; BO = burnout.

**Table 6:** Predictors of burnout (N = 219).

Variables		Burnout						95% Confidence Interval	
	*B*	*SE*	β	*T*	*p*	ES	VIF	Lower	Upper
(Constant)	36.11	2.55		14.15	<0.001 ***			31.08	41.14
Age	−0.09	0.05	−0.10	−1.71	0.089	0.24	1.76	−0.20	0.01
Education level	−0.42	0.63	−0.03	−0.67	0.504	0.09	1.04	−1.66	0.82
Job position	−1.33	0.81	−0.09	−1.63	0.104	0.22	1.74	−2.93	0.28
Job satisfaction at trauma center	2.84	0.67	0.20	4.23	<0.001 ***	0.57	1.13	1.51	4.16
Wish to continue working in a trauma center	−0.25	0.49	−0.02	−0.51	0.609	0.07	1.18	−1.21	0.71
TE	0.03	0.03	0.05	1.21	0.228	0.14	1.05	−0.02	0.09
CS	−0.50	0.04	−0.64	−13.06	<0.001 ***	1.69	1.28	−0.57	−0.42
SS	0.39	0.04	0.46	10.39	<0.001 ***	1.32	1.05	0.31	0.46
		adj *R*^2^ = 0.592, *F* = 40.60 (*p* < 0.001)							

Note: *** *p* < 0.001. Durbin-Watson’s du = 1.91 (du = 1.86, 4-du = 2.15); Breusch–Pagan’s χ^2^ = 0.00 (*p* > 0.999), Kolmogorov–Smirnov test (Z = 0.03, *p* = 0.950); Covariates (reference): education level (three-year college), job position (staff nurse), job satisfaction at trauma center (satisfied), wish to continue working in a trauma center (prefer). TE = traumatic event experience; CS = compassion satisfaction; SS = secondary traumatic stress; SE = standard error; ES = effect size (Cohen’s d); VIF = variance influence factor.

## Data Availability

The data presented in this study are available on request from the corresponding author and with permission from the Institutional Review Board of Eulji University.
